# A computational approach for detecting peptidases and their specific inhibitors at the genome level

**DOI:** 10.1186/1471-2105-8-S1-S3

**Published:** 2007-03-08

**Authors:** Lisa Bartoli, Remo Calabrese, Piero Fariselli, Damiano G Mita, Rita Casadio

**Affiliations:** 1 Laboratory of Biocomputing, CIRB/Department of Biology, University of Bologna, Bologna, Italy; 2 Department of Experimental Medicine, Biotechnology and Molecular Biology Section, Second University of Naples, Naples, Italy

## Abstract

**Background:**

Peptidases are proteolytic enzymes responsible for fundamental cellular activities in all organisms. Apparently about 2–5% of the genes encode for peptidases, irrespectively of the organism source. The basic peptidase function is "protein digestion" and this can be potentially dangerous in living organisms when it is not strictly controlled by specific inhibitors. In genome annotation a basic question is to predict gene function. Here we describe a computational approach that can filter peptidases and their inhibitors out of a given proteome. Furthermore and as an added value to MEROPS, a specific database for peptidases already available in the public domain, our method can predict whether a pair of peptidase/inhibitor can interact, eventually listing all possible predicted ligands (peptidases and/or inhibitors).

**Results:**

We show that by adopting a decision-tree approach the accuracy of PROSITE and HMMER in detecting separately the four major peptidase types (Serine, Aspartic, Cysteine and Metallo- Peptidase) and their inhibitors among a non redundant set of globular proteins can be improved by some percentage points with respect to that obtained with each method separately. More importantly, our method can then predict pairs of peptidases and interacting inhibitors, scoring a joint global accuracy of 99% with coverage for the positive cases (peptidase/inhibitor) close to 100% and a correlation coefficient of 0.91%. In this task the decision-tree approach outperforms the single methods.

**Conclusion:**

The decision-tree can reliably classify protein sequences as peptidases or inhibitors, belonging to a certain class, and can provide a comprehensive list of possible interacting pairs of peptidase/inhibitor. This information can help the design of experiments to detect interacting peptidase/inhibitor complexes and can speed up the selection of possible interacting candidates, without searching for them separately and manually combining the obtained results. A web server specifically developed for annotating peptidases and their inhibitors (HIPPIE) is available at

## Background

Peptidases (proteases) are proteolytic enzymes essential for the life of all organisms. The relevance of peptidases is proved by the fact that 2–5% of all genes encode for peptidases and/or their homologs irrespectively of the organism source [1]. In the SwissProt database [2] about 18% of sequences are annotated as "undergoing proteolytic processing", and there are over 550 known and putative peptidases in the human genome. It is also worth noticing that more than 10% of the human peptidases are under investigation as drug targets [3]. Proteases are responsible for a number of fundamental cellular activities, such as protein turnover and defense against pathogenic organisms. Since the basic protease function is "protein digestion", these proteins would be potentially dangerous in living organisms, if not fully controlled. This is one of the major reasons for the presence of their natural inhibitors inside the cell. All peptidases catalyze the same reaction, namely the hydrolysis of a peptide bond, but they are selective for the position of the substrate and also for the amino acid residues close to the bond that undergoes hydrolysis [4,5]. There are different classes of peptidases identified by the catalytic group involved in the hydrolysis of the peptide bond. However the majority of the peptidases can be assigned to one of the following four functional classes:

• Serine Peptidase

• Aspartic Peptidase

• Cysteine Peptidase

• Metallopeptidase

In the serine and cysteine types the catalytic nucleophile can be the reactive group of the amino acid side chain, a hydroxyl group (serine peptidase) or a sulfhydryl group (cysteine peptidase). In aspartic and metallopeptidases the nucleophile is commonly "an activated water molecule". In aspartic peptidases the side chains of aspartic residues directly bind the water molecule. In metallopeptidases one or two metal ions hold the water molecule in place and charged amino acid side chains are ligands for the metal ions. The metal may be zinc, cobalt or manganese, and a single metal ion is usually bound by three amino acid ligands [3]. Among the different ways to control their activity, the most important is through the interactions of the protein with other proteins, namely naturally occurring peptidase inhibitors. Peptidase inhibitors can or cannot be specific for a certain group of catalytic reactions. In general there are two kinds of interactions between peptidases and their inhibitors: the first one is an irreversible process of "trapping", leading to a stable peptidase-inhibitor complex; the second one is a reversible process in which there is a tight binding reaction without any chemical bond formation [4,6-8]. A shift of interest towards the mode of interaction of protein inhibitors with their targets is due to the possibility of designing new synthetic inhibitors. The research is driven by the many potential applications in medicine, agriculture and biotechnology.

In the last years, an invaluable source of information about proteases and their inhibitors has been made available through the MEROPS database [9], so that it is possible to search for known peptidase sequences (or structures) or peptidase-inhibitor sequences (or structures). Exploiting this source, in this paper we address the problem of relating a peptidase sequence (or inhibitor) with sequences that can putatively but reliably inhibit it (or proteases that can be inhibited by it). To this aim we implemented a method that first and reliably discriminates whether a given sequence is a peptidase or a peptidase-inhibitor, and afterwards gives a list of its putative interacting ligands (proteases/inhibitors). Our method provides answers to the following questions:

1) Given a pair of sequences, are they a pair of protease and inhibitor that can interact?

2) Given a protease (or inhibitor), can we predict the list of the proteins in a defined database that can inhibit (or be inhibited by) the query protein?

3) Given a proteome, can we compute the list of peptidases and their relative inhibitors for each protease class?

## Results and discussion

### Testing PROSITE and HMMER-Pfam capability of detecting MEROPS peptidases and inhibitors

The first step of our analysis is to evaluate the performance of PROSITE [10] on data sets of proteases and inhibitors, as derived from MEROPS [1,3,4,9]. Our method focuses on the four major classes of peptidases and their inhibitors as identified by the catalytic group involved in the hydrolysis of the peptide bond: Serine, Aspartic, Cysteine and Metallo- peptidases. In MEROPS there are annotations for 38 peptidase patterns and 20 inhibitor patterns. We adopted peptidases and inhibitors as annotated in MEROPS as the positive class (2793 peptidases and 1209 inhibitors). The negative counterpart was taken from PAPIA [11], and comprises non-inhibitor and non-peptidase non homologue sequences (2091 sequences) (see "Data sets" section). We start by running PROSITE on the PAPIA+MEROPS data sets. PROSITE can or cannot find a correct match. If a known inhibitor (peptidase) sequence is matched by a PROSITE inhibitor (peptidase) pattern we count it as a True Positive (TP), otherwise it is labeled as a False Negative (FN). Conversely, PAPIA sequences having a match with a PROSITE inhibitor (peptidase) pattern are False Positives (FP); otherwise they are True Negatives (TN).

In Table 1 the results obtained by filtering the PROSITE and the PAPIA+MEROPS data sets are listed. It is worth noticing that the PROSITE pattern search produces almost zero False Positives on the MEROPS+PAPIA data set, although with a significant number of False Negatives. This indicates that the method has a quite high specificity, but low coverage. In other words, a match has a high likelihood to be a true positive (high specificity); however due to the low coverage (61%, Table 1), still a non-match label may indicate a false negative (with a likelihood of 14% and 34% for inhibitors and peptidases, respectively).

In Table 2 we report the same type of analysis using HMMER-Pfam [12]. From the results it is evident that on average this method outperforms PROSITE. Our finding is in agreement with early observations indicating that Pfam is a better detection method than PROSITE [13]. We find that Pfam is more balanced than PROSITE, although with a slightly lower specificity (Table 1, 2).

### The decision-tree method

**Figure 1:**
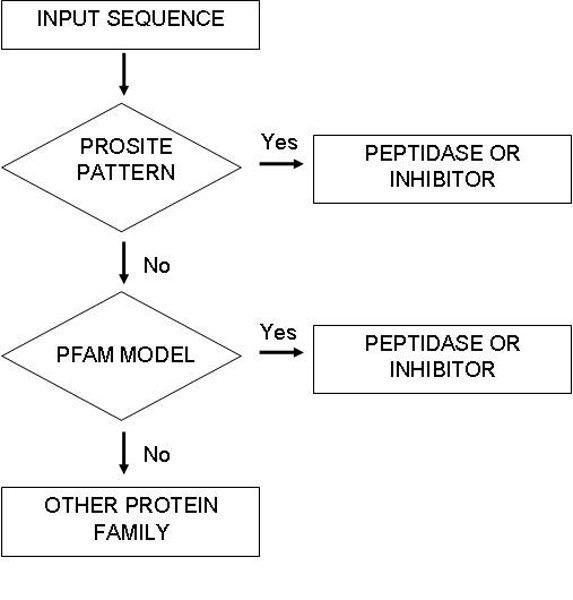
Flow-chart of the decision-tree method for the detection of peptidases and inhibitors.

The high level of PROSITE specificity prompted us to combine this pattern matching procedure with HMMER-Pfam by adopting a decision-tree method in order to take advantage of the features of both approaches (as described in Methods and shown in Figure 1). The results of the combined approach (as depicted into the flow chart of Figure 1) are then listed in Table 3. It appears that the overall performance is slightly improved over HMMER-Pfam alone. This is so particularly when the coverage of the positive class (Q [pos]) is considered.

### Detection of possible protease-inhibitor interacting pairs

The most relevant issue addressed by this paper is the measure of the detection accuracy of possible peptidase-inhibitor interacting pairs. The idea is to address questions related to the putative peptidase/inhibitor interaction (or combined discriminative efficacy). In order to test the combined accuracy of our decision-tree with respect to the PROSITE and HMMER-Pfam methods, we have taken all the possible sequence combinations of our selected data set, namely peptidase/inhibitor, peptidase/PAPIA, inhibitor/PAPIA, peptidase/peptidase, inhibitor/inhibitor, PAPIA/PAPIA, excluding the self-combinations (a sequence against itself). By adopting this procedure we ended up with 18,559,278 pairs that were scored as described below.

We divided MEROPS peptidase sequences in four classes according to their biological activity: Aspartic (A), Cysteine (C), Metallo (M) and Serine (S) peptidases. We labeled the inhibitors in the same way, with the exception that one more class is present for them, labeled as U; this set clusters all the inhibitors that are able to inhibit to some extent all types of peptidases (the so called Universal inhibitors).

**Figure 2:**
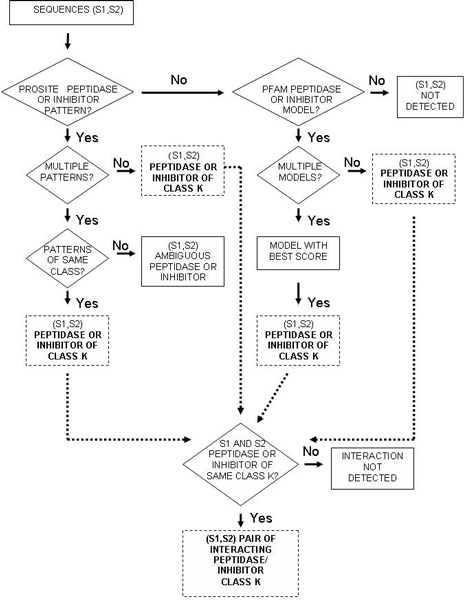
Flow-chart of the decision-tree method for the detection of possible peptidases/inhibitors interacting pairs. Each of the two input sequences is searched against Prosite and, in case of negative answer, against HMMER-Pfam. In both cases, when there is a match, the decision-tree method checks for the presence of multiple matches (patterns or models respectively). If there is a match, the method gives a positive answer for each sequence and only the peptidase and inhibitor sequences of the same class K (A, C, M, S, U) are classifed as possible interacting pairs.

Among the 18,559,278 possible pairs only those pairs pertaining to proteases and inhibitors of the same class are counted as members of the positive class (amounting only to 7 % of all possible pairs). All the remaining pairs are labeled as negative examples. On this data set we tested PROSITE, HMMER-Pfam and the combined decision-tree (Figure 2). We also tested the reverse decision-tree in which HMMER and PROSITE are swapped (alternative combinations are equivalent). In Table 4 it is shown that despite of the fact that the overall accuracy (Q2) is very high for all methods, the decision-tree outperforms all the others as the increased values of all scoring indexes indicate. Actually, the decision-tree approach shows the highest coverage and accuracy for both the peptidase-inhibitor interacting class and the negative set. It is also worth noticing that the correlation coefficient (C), that indicates the displacement from the random prediction, is very high for the decision-tree and it outperforms the second best method (HMMER) of 9 percentage points, with a false positive rate close to 0 (100-Q [neg]x100). This finding indicates that the decision-tree method can successfully be adopted to predict pairs of interacting peptidase/inhibitor, in order to sort out the subsets of possible interacting pairs of interest.

### Annotating peptidases and their inhibitors in Human and Mouse genomes

We applied the decision-tree method scored above to perform a large-scale genome annotation of peptidases and corresponding inhibitors of the Human and Mouse proteomes. We retrieved all known coding sequences and novel peptides from Ensembl35 [November 2005] [14]. The Human proteome consists of 33,869 sequences; the Mouse proteome contains 36,471 sequences. The decision- tree method is compared with PROSITE and HMMER-Pfam in singling out peptidases and inhibitors (Table 5 and 6, respectively). The predictive performance of the decision-tree method in predicting putative pairs of peptidase/inhibitor for each major class of both proteomes is reported in Table 7. Our results corroborate the view that among peptidases, the Aspartic class is less populated than the other three and this is so in both proteomes. For inhibitors, the less populated classes are Aspartic, Cysteine and Universal.

### Web server

In order to facilitate the user's search for protease/inhibitor interactions, we implemented a very simple web interface that exploits our developed decision-tree system. In practice it is possible to paste a sequence and the system checks whether that sequence is a protease or an inhibitor candidate. If the decision-tree returns a positive answer the server will provide the putative class among the four and the list of all possible known inhibitors (or proteases that might be inhibited by the query sequence). Furthermore, the web server furnishes also the corresponding lists of possible ENSEMBL protease-codes (or inhibitor-codes) of the Human and Mouse proteomes that belong to the predicted class of proteins and that can interact with the query sequence.

The server is available at [15].

## Conclusion

In this paper we developed a decision-tree based method that exploits the features of PROSITE and HMMER-Pfam in annotating peptidases and inhibitors and that is capable of correctly and reliably predict whether a given peptidase can or cannot interact with an inhibitor. The decision-tree discriminates peptidases or inhibitors with a score as high as 96% (97%) of correct predictions, improving both the coverage and the specificity of the positive class (pairs peptidase/inhibitor of the same class and pairs peptidase/Universal inhibitor) over PROSITE and HMMER-Pfam. Furthermore the decision-tree method is capable of predicting if a given protein pair is a pair of protease and inhibitor that can interact. This task can help in sorting out and speeding up the selection of possible interacting partners. Given a protease or an inhibitor the decision-tree method computes the list of the proteins in a defined database that can inhibit or that can be inhibited by the query protein. Finally, given a proteome the system provides the lists of peptidases and their relative inhibitors for each discriminated class.

## Methods

### The data sets

MEROPS database, hosted at the Sanger Institute [1,3,4], is the main resource of information on peptidases and their natural and synthetic inhibitors [9]. In this paper we refer to the 7.10 Merops release (22/07/2005) that contains 30909 peptidase sequences (including homologs) and 3690 inhibitor sequences (including homologs). We downloaded all data with the exclusion of sequences unassigned to any family. We then ended up with a set that contains chains of 167 protease families and 52 inhibitors families. We retained only the most abundant MEROPS functional classes: Serine, Aspartic, Cysteine and Metallo- peptidases.

From the MEROPS database we removed all sequences belonging to Threonin and Glutamic classes and the sequences of unknown catalytic type because for these groups no natural inhibitors are known. Our final peptidase set contains 2793 protein sequences. We also filtered out the inhibitor data set removing the family sequences that have an auto-inhibitory peptide at the N-terminus. Actually, these are peptidases with self-inhibitory peptides (I09 and I29 families). The inhibitor data set contains 1209 protein sequences. These two data sets represent the positive examples class for our classification method.

As a negative data set we have taken a non-redundant set of representative protein structures, of known function and not including peptidases and their inhibitors. This set was extracted from PAPIA (PArallel Protein Information Analysis system) [11]. The final PAPIA-derived set consists of 2091 protein chains.

### The decision-tree method

In order to predict if pairs of peptidase and inhibitor belong to the same class, we developed a system that performs two consecutive tasks: 1) extracts protease and inhibitor sequences from a given data set; 2) tests if they are compatible (if the inhibitor can interact with the protease). In order to solve this problem, we implemented a decision-tree method that processes the information obtained from PROSITE [10] and HMMER-Pfam [12,13] and detects if a query sequence could be annotated as peptidase or inhibitor. We selected PROSITE and Pfam since they are highly reliable methods for a classification task (see results).

PROSITE is a database of protein families and domains. It consists of biologically significant sites, patterns and profiles that help to reliably identify to which known protein family (if any) a new sequence belongs. We scanned all the data set against the PROSITE database (release 26/04/2005) with the "ps_scan" tool. Since we are interested in the detection of the presence/absence of patterns in the sequences, we used ps_scan for this task. We also set the options of skipping profiles and frequently matching patterns (unspecific) [10].

Pfam is a large collection of multiple sequence alignments and hidden Markov models covering many common protein domains and families [12]. Pfam is a database consisting of two parts, the first is the curated part of Pfam-A containing over 7,973 protein families, and the second is Pfam-B automatically generated for a more comprehensive coverage of known proteins. We downloaded a copy of the Pfam database (22/08/2005) and we used the HMMER package to search our protein sequence data set against the Pfam-A models. The Pfam library contains all local Pfam-A HMMs in a HMMER searchable format. We run the "hmmpfam" program to search for matches to a query sequence and the Pfam model of interest. The Pfam models annotated in MEROPS specific for our classes are 145, and 36 for proteases and inhibitors, respectively. If a sequence matches more than one model we consider the model with highest score and lowest e-value as the best.

The basic engine is described in the flow-chart of Figure 1, where for a given input sequence, we first look for PROSITE matching, and then in case of negative answer, we proceed using a profile-HMM scanning (HMMER-Pfam). From Figure 1, it is clear that if a PROSITE match is found, no more search is carried out. This works only if the first method has a high specificity (even when the sensitivity is low).

In order to predict whether a pair of sequences can be a peptidase and an inhibitor of the same class we run the decision-tree twice: first with the PROSITE and Pfam parameters relative to the peptidase search, and second adopting the model and the regular expressions corresponding to the inhibitors.

### Scoring indexes

All the results are evaluated using the following measures of efficiency. The fraction of correctly predicted residues is:

*Q2 *= *(TP+TN)/(TP+TN+FP+FN)*

where *TP *and *TN, FP *and *FN *are respectively: the number of true positives, true negatives, false positives and false negatives.

The correlation coefficient is defined as:

*cor *= [TP*TN - FP * FN]*/D*

where *D *is the normalization factor

*D *= [*(TP+FP)(TP+FN)(TN+FP)(TN+FN)*]^*1/2*^

The coverage or the sensitivity for the positive and negative classes is defined as:

*Q[pos] *= *TP/*[*TP+FN*]

*Q[neg] *= *TN/*[*TN+FP*]

The probability of correct predictions (accuracy or specificity) is computed as:

*P[pos] *= *TP/*[*TP+FP*]

*P[neg] *= *TN/*[*TN+FN*]

## Authors' contributions

All the authors contributed to the ideas and planning of this project. RC and LB carried out the analysis and wrote the software. Rita Casadio and PF supervised the study. GDM contributed to the inhibitors/peptidases analysis. PF, Rita Casadio, RC and LB contributed to the writing of this manuscript. All authors read and approved the final manuscript.

## Figures and Tables

**Table 1:** PROSITE discriminating capability towards MEROPS proteases and inhibitors.

***Data sets***	***Q2***	***Q [pos]***	***Q [neg]***	***P [pos]***	***P [neg]***	***C***
MEROPS (proteases)/PAPIA(sequences)	0.78	0.61	1	1	0.66	0.63
MEROPS (inhibitors)/PAPIA (sequences)	0.90	0.73	1	1	0.86	0.79

For definition see Scoring indexes

**Table 2:** HMMER-Pfam discriminating capability towards MEROPS proteases and inhibitors For definition see Scoring indexes.

***Data sets***	***Q2***	***Q [pos]***	***Q [neg]***	***P [pos]***	***P [neg]***	***C***
MEROPS (proteases)/PAPIA(sequences)	0.94	0.93	0.98	0.98	0.92	0.91
MEROPS (inhibitors)/PAPIA (sequences)	0.93	0.83	0.99	0.98	0.91	0.85

For definition see Scoring indexes

**Table 3:** Decision-Tree discriminating capability towards MEROPS proteases and inhibitors.

***Data sets***	***Q2***	***Q [pos]***	***Q [neg]***	***P [pos]***	***P [neg]***	***C***
MEROPS (proteases)/PAPIA(sequences)	0.96	0.93	1	1	0.91	0.92
MEROPS (inhibitors)/PAPIA (sequences)	0.97	0.94	0.99	0.99	0.97	0.95

For definition see Scoring indexes

**Table 4:** Scoring the detection of possible protease-inhibitor interactions with different methods.

***Methods***	***Q2***	***Q [pos]***	***Q [neg]***	***P [pos]***	***P [neg]***	***C***
Prosite	0.96	0.44	1	1	0.96	0.67
Hmm-Pfam	0.97	0.82	0.99	0.84	0.98	0.82
Decision-Tree	0.99	0.89	1	0.95	0.99	0.91
Reverse Decision-Tree	0.90	0.82	0.99	0.84	0.99	0.80

For definition of the statistical indexes see Scoring indexes

**Table 5:** Detection of proteases and inhibitors in the Human proteome.

	***Peptidases***						***Inhibitors***					
	
	***A***	***C***	***M***	***S***	***TOT***		***A***	***C***	***M***	***S***	***U***	***TOT***
***Prosite***	40	171	192	227	630		0	45	4	147	24	220
***Pfam***	164	575	626	698	2063		10	67	1099	446	52	1674
***Decision-tree***	183	600	654	735	2172		10	81	1099	501	68	1759

The different classes discriminated are: A = Aspartic-peptidase or inhibitor; C = Cysteine-peptidase or inhibitor; M = Metallo-peptidase or inhibitor; S = Serine-peptidase or inhibitor; U = Universal family of inhibitors.

**Table 6:** Detection of proteases and inhibitors in the Mouse proteome.

***Method***	***Peptidases***							***Inhibitors***					
	
	***A***	***C***	***M***	***S***	***TOT***			***A***	***C***	***M***	***S***	***U***	***TOT***
***Prosite***	96	181	234	242	753			0	59	4	171	21	255
***Pfam***	202	636	650	658	2146			16	84	1125	453	64	1742
***Decision-tree***	218	663	713	697	2291			16	91	1125	503	70	1805

For labels see Table 5.

**Table 7:** Detection of peptidase/inhibitor pairs in the Human and Mouse proteomes.

***Proteome***	***AA***	***CC***	***MM***	***SS***	***AU***	***CU***	***MU***	***SU***	***TOTAL***
***Human***	1830	48600	718746	368235	12444	40800	44472	49980	1285107 (0.2 %)*
***Mouse***	3488	60333	802125	350591	15260	46410	49910	48790	1376907 (0.2%)*

AA = Aspartic peptidase/Aspartic peptidase inhibitor pairs; CC = Cysteine peptidase/Cysteine peptidase inhibitor pairs; MM = Metallo-peptidase/Metallo-peptidase inhibitor pairs; SS = Serine peptidase/Serine peptidase inhibitor pairs; AU = Aspartic peptidase/Universal peptidase inhibitor pairs; CU = Cysteine peptidase/Universal peptidase inhibitor pairs; MU = Metallo-peptidase/Universal peptidase inhibitor pairs; SU = Serine peptidase/Universal peptidase inhibitor pairs.

* percentage of all the possible sequence pairs (573.537.646 and 665.048.685, for Human and Mouse genomes, respectively)
